# Immune Thrombocytopenia Induced by Immune Checkpoint Inhibitrs in Lung Cancer: Case Report and Literature Review

**DOI:** 10.3389/fimmu.2021.790051

**Published:** 2021-12-09

**Authors:** Wang Xie, NaNa Hu, LeJie Cao

**Affiliations:** Department of Pulmonary and Critical Care Medicine, The First Affiliated Hospital of University of Science and Technology of China, Division of Life Sciences and Medicine, University of Science and Technology of China, Hefei, China

**Keywords:** programmed cell death 1 inhibitor, immune thrombocytopenia, immune checkpoint inhibitor, atezolizumab, immune-related adverse event (irAE)

## Abstract

Immune checkpoint inhibitors (ICIs), including antibodies targeting programmed cell death protein-1 (PD-1) and programmed cell death ligand-1 (PD-L1), are being extensively used on advanced human malignancies therapy. The treatment with ICIs have acquired durable tumor inhibition and changed the treatment landscape in lung cancer. Immune-related adverse events including pneumonitis and thyroiditis have been well described, but less frequent events, such as ICIs-induced thrombocytopenia, are now emerging and may sometimes be severe or fatal. Since early detection and prompt intervention are crucial to prevent fatal consequences, it is of outmost importance that medical staff is aware of these potential toxicities and learn to recognize and treat them adequately. This review focuses on the epidemiology, clinical presentation, mechanisms, and clinical management of ICIs-induced thrombocytopenia in patients with lung cancer. We also present a patient with advanced lung adenocarcinoma who received the PD-L1 inhibitor atezolizumab and eventually developed severe thrombocytopenia. The case indirectly suggests that cytokine changes might contribute to immune dysregulation in ICIs-induced thrombocytopenia.

## Introduction

Immune checkpoint inhibitors (ICIs) lead to a significant improvement of overall survival (OS) in advanced non-small-cell lung cancer (NSCLC), and 8% of responded patients have achieved long-term survival (1). Atezolizumab, a humanized monoclonal antibody binding to programmed cell death-ligand 1, is currently approved for use against extensive small-cell lung cancer (SCLC) and has few treatment discontinuation rates due to adverse events (AEs) (2). Similar to other ICIs, atezolizumab can cause immune-related adverse events (irAEs), including endocrine, pneumonitis, hepatitis, thyroiditis, nephritis, and colitis. With the growing application of ICIs, the rate of hematologic toxicity has been observed in many cases (3, 4). In particular, ICIs-induced thrombocytopenia has been reported in a few cases with lung cancer (5). Herein, we reported a case of atezolizumab-induced immune thrombocytopenia and discussed the clinical management. We also review the epidemiology, clinical presentation, and prognosis of immune thrombocytopenia caused by ICIs in patients with advanced lung cancer.

## Case Presentations

**Figure 1:**
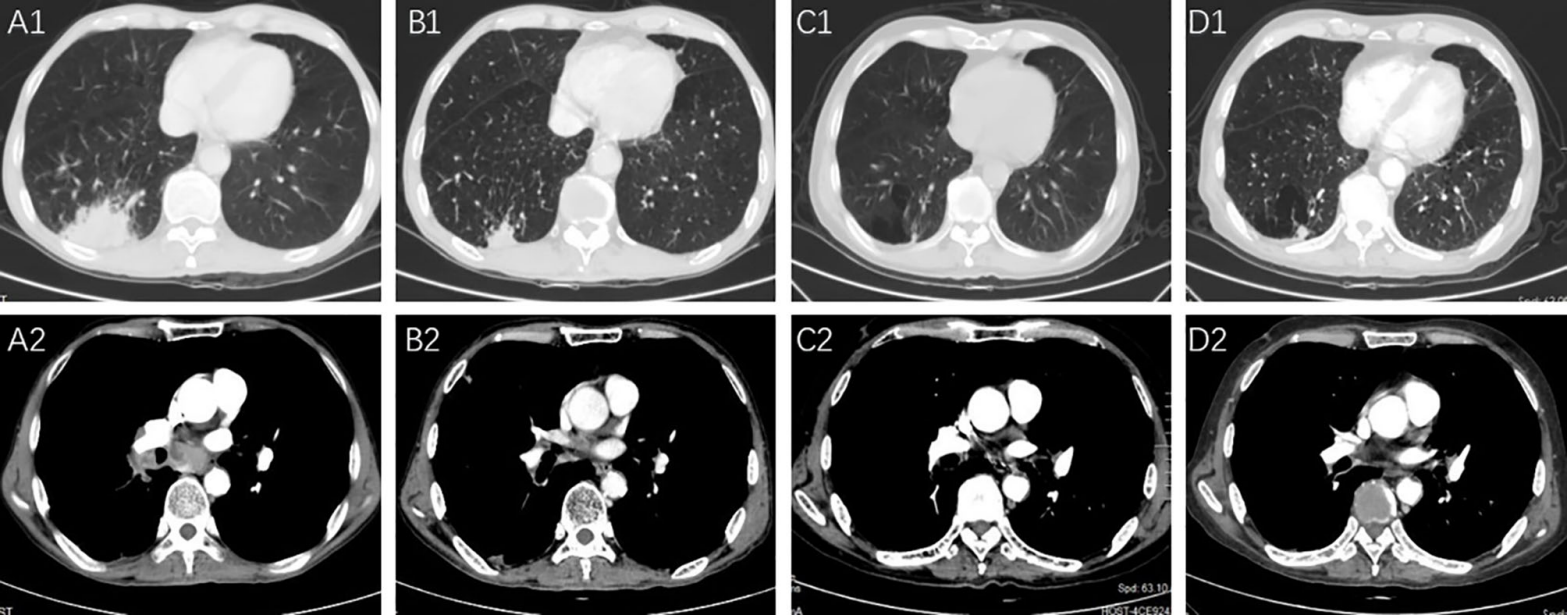
The pulmonary lesions and lymph node metastases before and after the PD-L1 treatment. **(A1, A2)** Right lower pulmonary lesions and swollen lymph nodes were revealed in the CT images before starting the chemotherapy in May 2018. **(B1, B2)** Partial response of the lesions in the right lower lobe and swollen lymph nodes was shown after 6 cycles of chemotherapy and atezolizumab treatment in August 2018; **(C1, C2)** the lesions showed continuous partial response after 36 cycles of pemetrexed and atezolizumab; **(D1, D2)** the lesions indicated a decreased size in March 2021.

**Figure 2:**
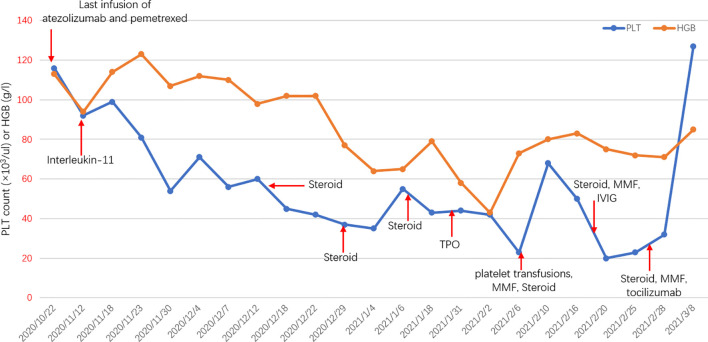
Longitudinal changes in PLT count or HGB over time. PLT, platelet; HGB, hemoglobin; MMF, mycophenolate mofetil; IVIG, intravenous immunoglobulin; TPO, thrombopoietin.

In a case report, a 76-year-old male with stage IV adenocarcinoma (cT2bN2M1b) without targetable genomic alterations, such as epidermal growth factor receptor (EGFR), anaplastic lymphoma kinase (ALK), rearranged c-ros oncogene 1 (ROS1), was diagnosed by right abdominal muscle surgical resection. Chest computed tomography (CT) revealed the tumor shadow in the right lower lobe of the lung, and it also demonstrated multiple swollen lymph nodes in the mediastinum (**Figure 1**). ^18^F-fluorodeoxyglucose positron emission tomography-computed tomography (PET/CT) showed multiple 18F-fluorodeoxyglucose (18F-FDG) uptake in the right abdominal muscle, L4, and right iliac bone. Baseline data showed that blood tests were normal, and Eastern Cooperative Oncology Group (ECOG) score was 1. The patient was recruited into a clinical study (IMpower 132) and received 6 cycles of carboplatin, pemetrexed, and atezolizumab (every 3 weeks) treatment. Then the patient showed a partial response indicated by a CT scan, and no severe toxicities were observed in February 2019, followed by 36 cycles of maintenance therapy with pemetrexed and atezolizumab. During the treatment, blood tests were performed every 3 weeks and no hematological abnormality was found. In November 2020, the patient developed thrombocytopenia (platelet level: 91×10^3^/ul) with normal hemoglobin and normal white cell counts and received the interleukin-11(IL-11) therapy to enhance the proliferation of megalokaryocytes for 2 weeks. Unfortunately, his platelet count slightly declined, and no autoimmune or coagulation disorders were displayed. As a result, the diagnosis was presumed as ICIs-induced thrombocytopenia, and he was treated with prednisone for 2 weeks (0.5mg/kg). However, his thrombocytopenia became worse with a sudden decrease in platelet level to 35×10^3^/ul. Thus, the pemetrexed and atezolizumab were discontinued. A bone marrow biopsy examination demonstrated no obvious morphological abnormalities, phagocytosis, or malignant invasion happened to this patient. Furthermore, antinuclear antibodies and other laboratory tests were negative, but antiphospholipid and antiplatelet antibodies were abnormal. After excluding chemotherapy, infection, pseudothrombocytopenia, or other drug-induced thrombocytopenia, atezolizumab-induced immune thrombocytopenia was finally diagnosed. Therefore, we gave him a high-dose steroid for 6 consecutive days and recombinant human thrombopoietin (TPO). However, his platelet count showed no improvement and stayed at the level of 23×10^3^/ul. He then received four times of platelet transfusions, mycophenolate mofetil, and infusion of intravenous immunoglobulin (IVIG), but his platelets did not recover. The lowest platelet level was recorded on February 20, 2021, with a level of 20×10^3^/ul. Interestingly, the level of serum interleukin-6 (IL-6) was significantly increased compared to the normal range, and we prescribed him an IL-6 receptor antagonist, tocilizumab, at 400 mg in addition to mycophenolate mofetil. Seven days later, his platelet counts started to increase and reach the normal range (100 to 300×10^3^/ul) by 1 week (**Figure 2**). The patient stayed a partial response to the treatment during atezolizumab therapy interruptions.

## Epidemiology of ICIs-Induced Thrombocytopenia

ICIs-induced thrombocytopenia in patients with lung cancer have been reported in many studies (**Table 1**), and thrombocytopenia has been demonstrated to be one of the most important hematological toxicity of hematological irAEs (21). There is usually a favorable prognosis in patients with thrombocytopenia. However, it is difficult to separate from chemotherapy-induced thrombocytopenia; therefore, the absolute incidence of ICIs-induced thrombocytopenia cannot be calculated. In one retrospective study, the incidence of ICIs-induced thrombocytopenia was less than 1% on ICIs (22), but in the observation study, the frequency of immune thrombocytopenia seems to be relatively high, accounting for 25–29% of hematological irAEs (23, 24). Besides, combination therapy was also associated with significantly higher risks of immune thrombocytopenia (5).

**Table 1:** Summary of reported lung cancer cases with immune thrombocytopenia after receiving ICIs.

Authors	Year	Tumor type	PD1/PD-L1	ICIs	Treatment lines	Radiotherapy	Onset times	Lowest PLT (×10^3^/ul)	Megakaryocyte	Treatment	Efficacy to ICIs	Outcome
Present case	2020	LAC	PD-L1	Atezolizumab	First	None	875 days	16	Normal	Steroids, TPO, platelet	PR	Recovered
										transfusions, MMF, tocilizumab		
Ito et al. (6)	2020	LAC	PD-1	Pembrolizumab	Second	None	11 days	0	Increased	Steroid, platelet	NA	Recovered
										transfusions, TRA, IVIG, rituximab		
Mori et al. (7)	2019	NSCLC	PD-1	Nivolumab	Second	None	15 days	2	Maintained	Steroids, platelet	PR	Recovered
										transfusions		
Hasegawa et al. (8)	2019	LAC	PD-1	Nivolumab	Second	Yes	42 days	2	ND	Steroids, platelet	NR	Died
										transfusions, IVIG, TRA		
Liu et al. (9)	2019	LAC	PD-L1	Durvalumab	First	None	266 days	12	Normal	Steroid, IVIG	PR	Died
Mouri et al. (10)	2019	LAC	PD-1	Pembrolizumab	First	Yes	21 days	0.3	Elevated	Steroid	PR	Improvement
Dickey et al. (11)	2019	LUSC	PD-1	Pembrolizumab	First	None	100 days	21	ND	Steroid	PR	Recovered
Song et al. (12)	2019	NSCLC	PD-1	Pembrolizumab	First	None	157 days	0	Normal	Steroid, IVIG, platelet	PR	Improvement
										transfusions, TRA		
Yılmaz et al. (13)	2019	LAC	PD-L1	Atezolizumab	Third	None	7 days	19	ND	Steroid, platelet	NA	Recovered
										transfusions, IVIG		
Suyama et al. (14)	2019	LUSC	PD-L1	Durvalumab	Third	Yes	28 days	7	Decreased	Steroid, platelet	PD	Recovered
										transfusions		
Tokumo et al. (15)	2018	LAC	PD-1	Nivolumab	Second	None	40 days	19	Absence	Steroids, platelet	PR	Died
										transfusions, IVIG		
Zhou et al. (16)	2018	LSCC	PD-1	Nivolumab	Third	None	180 days	0	Decreased	Steroid, plasma exchanges	SD	Recovered
Fu et al. (17)	2018	LAC	PD-1	Nivolumab	Third	Yes	255 days	11	Increased	Steroid, platelet	NA	Improvement
										transfusions, TPO, IVIG		
Jotatsu et al. (18)	2017	LAC	PD-1	Nivolumab	Third	None	29 days	16	Increased	Steroid	PR	Recovered
Karakas et al. (19)	2016	NSCLC	PD-1	Nivolumab	Second	None	85 days	5	Increased	Steroid, platelet	NA	Died
										transfusions		
Bagley et al. (20)	2016	LUSC	PD-1	Nivolumab	Second	Yes	99 days	33	NR	TRA	PR	Recovered

LAC, lung adenocarcinoma; NSCLC, non-small-cell lung cancer; LSCC, lung squamous cell carcinoma; LUSC, lung squamous cell carcinoma; ICI, immune checkpoint inhibitor; MMF, mycophenolate mofetil; IVIG, intravenous immunoglobulin; TRA, thrombopoietin receptor agonist; TPO, thrombopoietin; PR, partial response; CR, complete response; SD, stable disease; PD, progressive disease; NA, not available.

**Table 1** summarizes the reported cases of immune thrombocytopenia during ICIs therapy in patients with lung cancer. Most studies revealed that the onset of immune thrombocytopenia usually occurred within the first 12 weeks of ICIs initiation, but it can be at any time, even after cessation of treatment (25). Here, we reported the first case of one patient with lung cancer who developed late-onset immune thrombocytopenia during atezolizumab therapy. Similar to this case, delayed toxicity has been observed in other reports, which happened 1 to 2 years after monotherapy (26, 27). Generally, other irAEs have been shown to be associated with the efficacy of PD-1/PD-L1 inhibitors in patients with lung cancer (28, 29). Same as other irAEs, grade 1 thrombocytopenia during ICIs therapy is positively associated with Overall survival (OS), but lacks of Progression-free survival (PFS) benefit (30). Immune thrombocytopenia usually is not fatal, but deaths caused by the adverse events had been reported in uncommon cases (25). Thus, ICIs-induced thrombocytopenia is potentially life-threatening and should be paid close attention in clinical practice.

## Mechanism of ICIs-Induced Thrombocytopenia

Although the mechanism of drug-induced thrombocytopenia has been well demonstrated, the underlying mechanism of ICIs-induced thrombocytopenia remains unclear (31). Reinvigoration of exhausted CD4^+^ helper T cells and CD8^+^ cytotoxic T cells activates inflammatory pathways and ultimately results in damage to hematopoietic stem cells. In addition, ICI-induced antiplatelet antibody production can promote platelet destruction, which is supported by high antiplatelet antibody levels in patients who suffered from ICIs-induced thrombocytopenia (7, 18). Furthermore, the expression of PD-L1 on platelets in lung cancer patients was significantly increased, which might render susceptible targets of antibody-based anti-PD-L1 therapies. As a result, the amount of PD-L1-expressing platelets dramatically decreased in the blood of patients receiving PD-L1 therapy (32). Another suggested mechanism was activation of T-cells, leading to the secretion of different cytokines from T-helper cells (33). In the present case, although the patient has a high level of platelet-associated immunoglobulin G antibody and other possible causes of thrombocytopenia, particularly a viral infection, were excluded and multiple treatments were used including steroid, platelet transfusion, IVIG, and mycophenolate mofetil, the platelet counts recovered slightly. Importantly, the serum level of IL-6 was significantly higher than that of the normal range, indicating that the potential mechanism of ICIs-induced thrombocytopenia was relying on the abnormal cytokine secretion of activated lymphocytes. Consequently, the IL-6 receptor antagonist tocilizumab has achieved a partial response.

## Diagnosis and Treatment

ICIs-induced thrombocytopenia mimics virtually any other type of thrombocytopenia, making it a diagnosis of exclusion and is difficult due to the lack of specific testing. Any new platelet count decrease should be considered as immune thrombocytopenia for patients receiving ICIs therapy. Generally, bone marrow examination is necessary to exclude dysplasia or cancer invasion. In addition, other examinations should be administrated to distinguish ICIs-induced thrombocytopenia from other etiologic agents, such as drug-induced thrombocytopenia (heparin/HITT, chemotherapy, etc.), infections, hematological malignancies (myelodysplastic syndrome, etc.), platelet sequestration (spleen, liver diseases), platelet consumption (thrombotic thrombocytopenic purpura, etc.) (9, 34, 35). Furthermore, the presence of a high platelet-associated IgG titer may be helpful to diagnose ICIs-induced thrombocytopenia (14). Therefore, effective recognition and diagnosis for immune thrombocytopenia are important because of the different prognosis and therapeutic management. In our case, absence of liver, spleen, or rheumatologic disease; malignant infiltration of the bone marrow; and other causes of thrombocytopenia suggest that thrombocytopenia was induced by atezolizumab. Sometimes, the lack of efficacy of transfusions during and after ICI administration is indicative of ICIs-induced thrombocytopenia.

The targeted therapies are not well defined for immune thrombocytopenia induced by ICIs. According to the American Society of Clinical Oncology (ASCO) guidelines, grading treatment on severity classification is the current principle for immune thrombocytopenia (36). Generally, the management of grade 1 toxicities (<100×10^3^/ul) should comply with the clinical and laboratory evaluation. Sometimes ICIs need not stop, and platelet changes should be continued with close monitoring. Withholding ICIs therapy is generally recommended for grade 2 toxicities (<75×10^3^/ul) until the platelets recover to grade 1 toxicities, and oral corticosteroids (0.4–1 mg/kg/day of prednisone or equivalent) should be presented with 2–4 weeks and/or conjunctive use of IVIG. But the dose adjustment is not advised if ICIs therapy is re-administrated because irAEs are not dose-dependent (37). For grade 3 toxicities (<50×10^3^/ul) or grade 4 toxicities (<25×10^3^/ul), ICIs therapy must be definitively discontinued until return to grade 1 toxicities, with the administration of high-dose corticosteroids and optional IVIG, with permanent ICIs, withdrawal is necessary if platelet do not resolute to normal. Other therapy strategies include recombinant human TPO, romiplostim, platelet transfusion, and immunosuppressive agents, such as azathioprine and rituximab. Steroids are generally essential for treating immune thrombocytopenia by ICIs but are not always effective in severe thrombocytopenia. Many studies have demonstrated that the presence of specific single-nucleotide polymorphisms, such as PD-1 -606 AA genotype and +63379 TT genotype, affects the susceptibility to prednisolone treatment (38). In addition, HLA-DRB1*0410 or HLA-DRB1*0405 allele in the patients’ immune thrombocytopenia has been reported to contribute to steroid therapy resistance (20, 39). This can explain why the patient had a weak response to steroid therapy.

## Conclusion

Although ICIs-induced thrombocytopenia is rare in patients receiving ICIs, we still need to pay more attention to this issue because of its life-threatening characteristic. Any new abnormality of platelet counts should be considered as a potential clinical significance for immunotherapy patients; thus, careful recognition and accurate diagnosis are extremely important. Although its response to steroids, IVIG, and platelet transfusion is relatively good, the underlying mechanism of immune thrombocytopenia remains elusive, and further study is awaited.

## Author Contributions

LC developed the idea for the study. WX and NH did data collection, data analysis, and manuscript preparation. All authors have reviewed and approved the final version of the manuscript and have consented to its publication.

## Funding

This work was supported by the National Natural Science Foundation of China (82000082), Natural Science Foundation of Anhui Province (2008085QH353), and the Fundamental Research Funds for the Central University (WK9110000124).

## Conflict of Interest

The authors declare that the research was conducted in the absence of any commercial or financial relationships that could be construed as a potential conflict of interest.

## Publisher’s Note

All claims expressed in this article are solely those of the authors and do not necessarily represent those of their affiliated organizations, or those of the publisher, the editors and the reviewers. Any product that may be evaluated in this article, or claim that may be made by its manufacturer, is not guaranteed or endorsed by the publisher.
